# Supplementary material for: End Sequence Analysis Toolkit (ESAT) expands the extractable information from single-cell RNA-seq data
Source: Genome Res. 2016 Oct;26(10):1397–410. doi: 10.1101/gr.207902.116 (PMC5052061; doi:10.1101/gr.207902.116)
Supplement: Supplemental Material [file supp_gr.207902.116_Supp_Materials.tar.gz › ESAT-esat_v0.1/doc/html/interfacebroad_1_1core_1_1annotation_1_1_annotation_factory_3_01_t_01extends_01_lightweight_genomic_annotation_01_4.html]

ESAT: broad.core.annotation.AnnotationFactory< T extends LightweightGenomicAnnotation > Interface Reference


ESAT


- Main Page
- Related Pages
- Packages
- Classes
- Files

- Class List
- Class Index
- Class Hierarchy
- Class Members

All Classes Namespaces Files Functions Variables Enumerator Pages

- broad
- core
- annotation
- AnnotationFactory< T extends LightweightGenomicAnnotation >

Public Member Functions |
List of all members

broad.core.annotation.AnnotationFactory< T extends LightweightGenomicAnnotation > Interface Reference

Inheritance diagram for broad.core.annotation.AnnotationFactory< T extends LightweightGenomicAnnotation >:

[legend]

Collaboration diagram for broad.core.annotation.AnnotationFactory< T extends LightweightGenomicAnnotation >:

[legend]

|  |  |
| --- | --- |
| Public Member Functions | |
| T | create (String[] rawFields) throws ParseException |
|  | |
| T | create (GenomicAnnotation baseAnnotation) |
|  | |
| T | create (String name) |
|  | |

## Member Function Documentation

|  |  |  |  |  |  |
| --- | --- | --- | --- | --- | --- |
| T broad.core.annotation.AnnotationFactory< T extends LightweightGenomicAnnotation >.create | ( | String[] | *rawFields* | ) | throws ParseException |

|  |  |  |  |  |  |
| --- | --- | --- | --- | --- | --- |
| T broad.core.annotation.AnnotationFactory< T extends LightweightGenomicAnnotation >.create | ( | GenomicAnnotation | *baseAnnotation* | ) |  |

|  |  |  |  |  |  |
| --- | --- | --- | --- | --- | --- |
| T broad.core.annotation.AnnotationFactory< T extends LightweightGenomicAnnotation >.create | ( | String | *name* | ) |  |

---

The documentation for this interface was generated from the following file:

- java/broad/core/annotation/AnnotationFactory.java


---

Generated on Fri Aug 15 2014 15:10:50 for ESAT by  

 1.8.7
